# Draft Genome Sequence of *Microvirga vignae* Strain BR 3299^T^, a Novel Symbiotic Nitrogen-Fixing Alphaproteobacterium Isolated from a Brazilian Semiarid Region

**DOI:** 10.1128/genomeA.00700-15

**Published:** 2015-07-09

**Authors:** Jerri Edson Zilli, Samuel Ribeiro Passos, Jakson Leite, Gustavo Ribeiro Xavier, Norma Gouvea Rumjaneck, Jean Luiz Simoes-Araujo

**Affiliations:** a Embrapa Agrobiologia, Seropedica, Brazil; b Universidade Federal Rural do Rio de Janeiro, Seropedica, Brazil

## Abstract

*Microvirga vignae* is a recently described species of root-nodule bacteria isolated from cowpeas grown in a Brazilian semiarid region. We report here the 6.4-Mb draft genome sequence and annotation of *M. vignae* type strain BR 3299. This genome information may help to understand the mechanisms underlying the ability of the organism to grow under drought and high-temperatures conditions.

## GENOME ANNOUNCEMENT

*Microvirga vignae* strain BR 3299^T^ was isolated from a cowpea root nodule growing in a soil sample collected from a Brazilian semiarid region (1). Most bacteria isolated from cowpea nodules cultivated in this region are not taxonomically characterized, although they are potentially members of *Microvirga* (2, 3). Drought stress is one of the major harsh environmental conditions affecting rhizobium symbiosis in semiarid lands. Furthermore, soil temperatures near the surface can be very high, and although they decrease rapidly with increasing depth, being around 30 to 35°C at 15 cm, the negative impact on legume nodule formation and dinitrogen fixation can be significant for several species (4). Here, we report the draft genome sequence of strain BR 3299, a type strain of *M. vignae* and a symbiotic nitrogen-fixing member of the *Alphaproteobacteria*.

BR 3299^T^ total genomic DNA was extracted and purified (5) from a culture grown in yeast mannitol agar (YMA) medium. The whole genome was sequenced using the 100-bp paired-end Illumina MiSeq system (Macrogen, South Korea), yielding a total of 11,703,138 reads, which corresponds to approximately 181× coverage sequencing. The read quality was analyzed using FastQC (http://www.bioinformatics.babraham.ac.uk/projects/fastqc) and trimmed using the FASTX-Toolkit, and only bases with a quality score of >20 (Q20) were used. *De novo* assembly was performed using CLC Genomics Workbench 7 (CLC bio, USA), and contigs of <400 bp were eliminated.

The annotation and identification of metabolic pathways for the draft genome sequence were carried out using the RAST version 2.0 server (6). tRNAs were predicted with tRNAscan-SE 1.23 (7) and rRNAs were predicted with RNAmmer 1.2 (8). The KEGG pathways and Pfam annotation were analyzed using WebMGA (9). The annotated genome was submitted to the Joint Genomics Institute (IMG/ER database) (https://img.jgi.doe.gov/cgi-bin/er/main.cgi) which was used to refine the annotation and registration at the International Nucleotide Sequence Database Collaboration (GenBank, USA).

The draft genome sequence of *M. vignae* strain BR 3299^T^ consists of 165 contigs and includes 6,472,445 bp, with an overall G+C content of 61%. In the genome, 6,641 protein-coding sequences (CDSs), distributed in 451 subsystems, as well as 60 copies of RNA were predicted. Approximately 62.7% of the CDSs were assigned specific functions using RAST annotation, and a Pfam reference family was identified for 36% of the CDSs, whereas KEGG orthology (KO) numbers were assigned for 65%. All enzymes required for biological nitrogen fixation are encoded by the genome. Furthermore, other genes involved with nitrogen metabolism, including three genes for nitrosative stress, 11 for ammonia assimilation, and 26 genes related to denitrification were identified. A large number of genes related to stress response were also identified, including 80 genes for oxidative stress, 24 genes for osmotic stress, and 18 genes encoding heat shock proteins. This genomic information may help us to understand the mechanisms underlying the ability of the bacterium to grow and fix nitrogen in symbiosis under drought and high-temperature conditions.

### Nucleotide sequence accession number. 

This whole-genome shotgun project has been deposited at DDBJ/EMBL/GenBank under the accession no. LCYG00000000 (BioProject no. PRJNA283551). The version described in this paper is the first version.
